# Supplementary material for: Si-C is a method for inferring super-resolution intact genome structure from single-cell Hi-C data
Source: Nat Commun. 2021 Jul 16;12:4369. doi: 10.1038/s41467-021-24662-z (PMC8285481; doi:10.1038/s41467-021-24662-z)
Supplement: Supplementary file 1 — Supplementary Information [file 41467_2021_24662_MOESM1_ESM.pdf]

Supplementary Information

Si-C method for inferring super-resolution intact genome structure from single-cell Hi-C data

Luming Meng[1*], Chenxi Wang[2], Yi Shi[3] and Qiong Luo[2]

[1]MOE Key Laboratory of Laser Life Science & Guangdong Provincial Key Laboratory of Laser Life Science, College of Biophotonics, South China Normal University, Guangzhou 510631, China
[2]Center for Computational Quantum Chemistry, School of Chemistry, South China Normal University, Guangzhou 510631, China
[3]Bio-X Institutes, Key Laboratory for the Genetics of Developmental and Neuropsychiatric Disorders, Shanghai Jiao Tong University, 1954 Huashan Road, Shanghai 200030, China

*Corresponding author: menglum@scnu.edu.cn

Table of contents

Supplementary Note 1. The process of calculating 3D genome structure by NucDynamics

Supplementary Note 2. The process of calculating 3D genome structure by SCL

Supplementary Note 3. Computing resources

Supplementary Note 4. Validating inferred 3D structures using experimental Fluorescence *in situ* hybridization (FISH) data

Supplementary Note 5 Calculating root mean square deviation (RMSD)

Supplementary Note 6. Translating calculated 3D genome structure to distance matrix

Supplementary Note 7. Identifying boundaries of domain structures in distance matrix and boundaries of TADs in the populated Hi-C data

Supplementary Note 8. Calculation of chromosome intermingling

Supplementary Note 9. Identification of A/B compartment and features of large-scale 3D structure of the genome

Supplementary Note 10. Calculating gyration radius for chromatin region

Supplementary Table 1. Source list of experimental data and pre-calculation

Supplementary Figure 1. Correlations between distances measured by FISH probes and distances in the published NucDynamics 100-kb structures and in the Si-C 100-kb structures.

Supplementary Figure 2. Comparison of pairwise RMSD for different structure ensembles.

Supplementary Figure 3. Plot of the percentage of loops that are formed in the Si-C inferred structures of 10-kb resolution.

Supplementary Figure 4. Cross-sections of the Si-C intact genome 3D 10-kb resolution structures of Cell 2-8.

Supplementary Figure 5. Violin plot showing the proportion of each chromosome that intermingles with other chromosomes.

Supplementary Figure 6. The locations of centromeres and telomeres in the Si-C intact genome 3D 10-kb resolution structures for the all eight individual cells.

Supplementary Note 1. The process of calculating 3D genome structure by NucDynamics

To reconstruct 3D genome structure ensemble of cell 1 for the comparisons shown in Fig. 2b and 2c, we downloaded the source code of NucDynamics from the website https://github.com/TheLaueLab/nuc_dynamics. For the calculation to generate a 10-kb resolution structure ensemble including 20 conformations, we executed the NucDynamics software by the command as follows:

./nuc_dynamics Cell_1_contact.ncc –m 20 –f pdb –o Cell_1_10kb_20replica.pdb –s 10.24 5.12 2.56 1.28 0.64 0.32 0.16 0.08 0.04 0.02 0.01 –cpu 20

where Cell_1_contact.ncc is the Hi-C data of Cell 1 that is the exactly same data used for the structure determination of the Si-C method. Because a hierarchical protocol is employed in the NucDynamics framework, calculations were performed at 10240-kb, 5120-kb, 2560-kb, 1280-kb, 640-kb, 320-kb, 160-kb, 80-kb, 40-kb, 20-kb and finally 10-kb resolution. The values in the list "10.24 5.12 2.56 1.28 0.64 0.32 0.16 0.08 0.04 0.02 0.01" in the command represents the mentioned resolutions. For instance, the value of 10.24 means 10240-kb resolution and the last value in the list, 0.01, means the resolution of final output structure is 10 kb. "Cell_1_10kb_20replica.pdb" in the command is the name of the output file which includes 20 calculated structure replicas of 10-kb resolution. In the same manner, we generated 20-kb structure ensemble including 20 conformations by the command as follows:

./nuc_dynamics Cell_1_contact.ncc –m 20 –f pdb –o Cell_1_20kb_20replica.pdb –s 10.24 5.12 2.56 1.28 0.64 0.32 0.16 0.08 0.04 0.02 –cpu 20

, and so on for other resolution structure ensemble calculations.

Supplementary Note 2. The process of calculating 3D genome structure by SCL

We use single-cell lattice (SCL) method to reconstruct 3D structure of chromosome 1 of cell 1. The code is downloaded from website http://dna.cs.miami.edu/SCL/. The running command is

./scl -I ../data/ES_Cell1_chr1.txt -o ../output/ES_cell/chr1/100kb/ES_Cell1_chr1_model -res 0.1

Supplementary Note 3. Computing resources

Cpus of Intel(R) Xeon(R) CPU E5-2692 v2 @ 2.20GHz were used to compare Si-C, NucDynamics and SCL in terms of computation cost.

Supplementary Note 4. Validating inferred 3D structures using experimental Fluorescence *in situ* hybridization (FISH) data

Beagrie *et al.* used eight FISH probe pairs which are located on chromosomes 3 and 11 of mESC cells to detect the spatial distances between the regions that are hybridized by a probe pair. The numbers of sample cells measured by the eight probe pair range from 26 to 119. The median distance of each pair is chosen to assess the validity of inferred structures. To achieve the aim, we first identified the beads in inferred structures that are corresponding to the hybridized regions of each pair. In this study, at a given resolution, we generate a structure ensemble including 20 conformations for each individual mESC cell. There are eight mESC individual cells under investigation. Therefore, for each probe pair, we can obtain a total number of 160 distances from inferred structures at a given resolution, and the median value is used to calculate the correlation with the median spatial distances measured by the FISH experiment. The correlations for the Si-C structures of 10-kb resolution and 100-kb resolution, as well as the Nucdynamic structures of 100-kb are calculated, and the Pearson correlation coefficients are 0.889, 0.931, and 0.888, respectively.

Supplementary Note 5. Calculating root mean square deviation (RMSD)

Before assessing the variability within the 3D genome structure ensemble calculated from sparse single-cell Hi-C data, it should be noted that the reconstructed structures are not well defined, since there are some genome regions within which no contacts were detected by the single-cell Hi-C experiments of all 8 cells. We named such regions as void regions. A brief description of the process of identifying void regions is the following. First, we divided chromosomes into beads representing 800-kb region of chromosome sequence. Second, we mapped contact reads derived from the all 8 Hi-C datasets to the beads and identified the beads where no contacts are observed as void regions. Models of 400-kb, 200-kb, and 100-kb resolutions share void regions with the 800-kb resolution model. In the same way, we identified void regions in 640-kb and 512-kb resolution models. Therefore, models of (320-kb, 160-kb, 80-kb, 40-kb, 20-kb and 10-kb) resolutions and (56-kb, 128-kb, 64-kb, 32-kb, 16-kb, 8-kb, 4-kb, 2-kb and 1-kb) resolutions share the void regions with the 640-kb and 512-kb resolution models, respectively. The void regions were excluded from the analyses of structural variability between the ensemble members.

Root mean square deviation (RMSD) is widely used to measure structural variability. In this study, we calculated RMSD between conformations within a structural ensemble according the

algorithm reported by Theobald[1], where the RMSD between two conformations is defined as:

$$RMSD = \min_{trans+rot}\{\sqrt{\frac{1}{n}\sum_{i=1}^{n}(\vec{r}_{i,1} - \vec{r}_{i,2})^2}\} \quad \text{(S1)}$$

in which $\vec{r}_{i,1}$ and $\vec{r}_{i,2}$ are the coordinates of the ith bead of the two conformations, n is the total number of beads taken into account for the RMSD calculation. The value of RMSD is a minimum value obtained by optimally aligning the two conformations through translation and rotation.

One problem of structure reconstruction from Hi-C data is that misreconstruction such as mirror images can not be distinguished by the Hi-C experiment. Although the conformation appears in the same spatial folding as its mirror image, the RMSD between them would be high. Therefore, the variability within the ensemble including image-mirror conformations will be seriously overestimated. To overcome the issue, when calculating the pairwise RMSD between pair of conformations, denoted as Conformations (1, 2) (the numbering is quite arbitrary), we firstly constructed a image-mirror structure for Confromation 1, denoted as Conformation $1'$ and then calculated RMSD twice, one for Conformations (1, 2), and the other for Conformations ($1'$, 2). The smaller RMSD is retained to describe the variability between Conformation (1, 2).

For convenience of the comparisons displayed in Fig. 2d, we set nuclear radius to the unity of RMSD based on the implicitly assume that intact genome 3D structure of each cell investigated here is a sphere of the same size. The nuclear radius is defined as the maximum spatial distance (in the unit of bead diameter) among the distances between every bead and the centroid of all beads.

Code for calculating RMSD is available at:

https://github.com/TheMengLab/Si-C/tree/master/analysis/structure_analysis/analysis/align/rmsd

Supplementary Note 6. Translating calculated 3D genome structure to distance matrix

For each calculated 3D genome structure, one can measure the spatial distance between each pair of beads in the 3D structure and translate the structure into a distance matrix where matrix element represent the spatial distance between corresponding beads in the 3D genome structure. It should be noted that the value of matrix element of each distance matrix shown in Fig. 2f and 3a is computed by averaging the distance between each pair of beads across the whole 20 members of the same structure ensemble.

Supplementary Note 7. Identifying boundaries of domain structures in distance matrix and boundaries of TADs in the populated Hi-C data

Separation score is used to quantify the degree of separating the upstream and downstream chromatin regions of one specific sequence position. The separation score is calculated from the distance matrix. Specifically, the separation score of each position is computed by averaging all the spatial distances between any pair of positions separately located in the two 500 kb regions on either side of the position. Code for Separation Score calculation is available at: https://github.com/TheMengLab/Si-C/tree/master/analysis/structure_analysis/analysis/align/sepscore_gyr

The positions that are identified as boundaries of domain structures in distance matrix should stratify two criteria. First, the boundary position should have higher separation score than any other positions within the 400 kb regions on either side of the position. Second, the separation score of each position within the region under investigation can be calculated and their average separation score can be obtained immediately. The boundary positon should be higher than the average separation score. The code for identifying boundary position in distance matrix is available at: https://github.com/TheMengLab/Si-C/tree/master/analysis/structure_analysis/analysis/align/sepscore_gyr/boundary_chr

The population Hi-C data shown in Fig. 3c and 3e is downloaded from Gene Expression Omnibus (GEO) repository with accession code GSE35156. The process for the identification of TAD boundaries in the populated Hi-C data includes the following steps:

(1) Converting the reference genome of Hi-C data from NCBI37/mm9 to GRCm38/mm10.

(2) All chromosome chains are divided into beads of 10-kb size and all Hi-C contact reads are assigned to pairs of beads containing the corresponding restriction fragment ends. After mapping, Hi-C data is presented in three columns, one of which lists the count of contact reads and the other two columns display the genome positions corresponding to the restriction fragment ends of contact reads.

(3) Normalizing the Hi-C data by using iterative correction and eigenvector decomposition (ICE) algorithm. The Hi-C data for individual chromosome is normalized, respectively.

(4) Converting the format of normalized Hi-C data from the form of three columns to the matrix format.

(5) Using TopDom method[3] (version 0.0.2) to identify the TAD boundaries in each chromosome. A window size of 10 is used in the identification process.

Supplementary Note 8. Calculation of chromosome intermingling

To assess the degree of intermingling between chromosomes, we first identified intermingled beads within each chromosome and then calculated the proportion of intermingled beads to the total beads of the chromosome. The intermingled beads were defined as those that surrounded by at least four other beads from a different chromosome within a distance threshold between beads of 2 bead diameters.

Supplementary Note 9. Identification of A/B compartment and features of large-scale 3D structure of the genome

The identification of chromosome compartment is calculated following a similar algorithm described in the previous work [4]. In the calculation process, we first normalized the Hi-C contact frequency matrix through dividing each matrix element by the genome-wide average contact frequency for bin pairs at the same genomic distance. Then we calculated the correlation matrix **M**, in which the element M_{ij} describes the Pearson correlation between the ith and j^{th} rows of the normalized Hi-C matrix generated in the first step. Based on the correlation matrix **M**, each chromosome was partitioned into two types of regions according to the first principal component generated by principal component analysis. Between these two types of regions, the one with higher overlap with the H3K4me3 enriched regions was defined as compartment A and the other one was defined as compartment B.

The script is available at: https://github.com/TheMengLab/Si-C/tree/master/analysis/compartment

Supplementary Fig.2 displays several features of 3D genome architecture for Cells 2-8. Supplementary Fig.3 shows the locations of centromeres and telomeres in the nucleus for the all eight cells.

Supplementary Note 10. Calculating gyration radius for chromatin region

We estimated the degree of compaction of investigated regions of 200 kb using the gyration radius (R_g) which is defined as the following:

$$R_g = \sqrt{\frac{1}{N}\sum_{i=1}^{N}(\vec{r}_i - \vec{r}_{ave})^2} \qquad \text{(S2)}$$

in which N represents the number of beads in the 200 kb region under investigation, $\vec{r}_i$ the

coordinate of the ith bead in the region and $\vec{r}_{ave}$ is the coordinate of the centroid of the region. In this study, the value of N is 20 because the region of 200 kb is represented by beads of 10-kb size.

The script for calculating is gyration radius available at: https://github.com/TheMengLab/Si-C/tree/master/analysis/structure_analysis/analysis/align/sepscore_gyr

Supplementary Table 1. Source list of experimental data and pre-calculation

The experimental data used in our analysis was taken from previously published work, as elaborated below:

Data type	Accession number	Reference
H3K4me3 ChIP-seq (haploid)	GSE80280	Stevens, T.J. et al. 3D structures of individual mammalian genomes studied by single-cell Hi-C. *Nature* **544**, 59-+ (2017).
Constitutive Lamina Associated Domain	GSE17051	Peric-Hupkes, D. et al. Molecular Maps of the Reorganization of Genome-Nuclear Lamina Interactions during Differentiation. *Mol Cell* **38**, 603-613 (2010).
Replication Timing	E-MTAB-3506	Kolesnikov, N. et al. ArrayExpress update-simplifying data submissions. *Nucleic Acids Res* **43**, D1113-D1116 (2015).
Populated Hi-C data	GSE35156	Dixon, J.R. et al. Topological domains in mammalian genomes identified by analysis of chromatin interactions. *Nature* **485**, 376-380 (2012).
Loop anchors		Dixon, J.R. et al. Topological domains in mammalian genomes identified by analysis of chromatin

		interactions. *Nature* **485**, 376-380 (2012).
3D FISH data		Beagrie, R. A. *et al.* Complex multi-enhancer contacts captured by genome architecture mapping. *Nature* **543**, 519-+, doi:10.1038/nature21411 (2017).

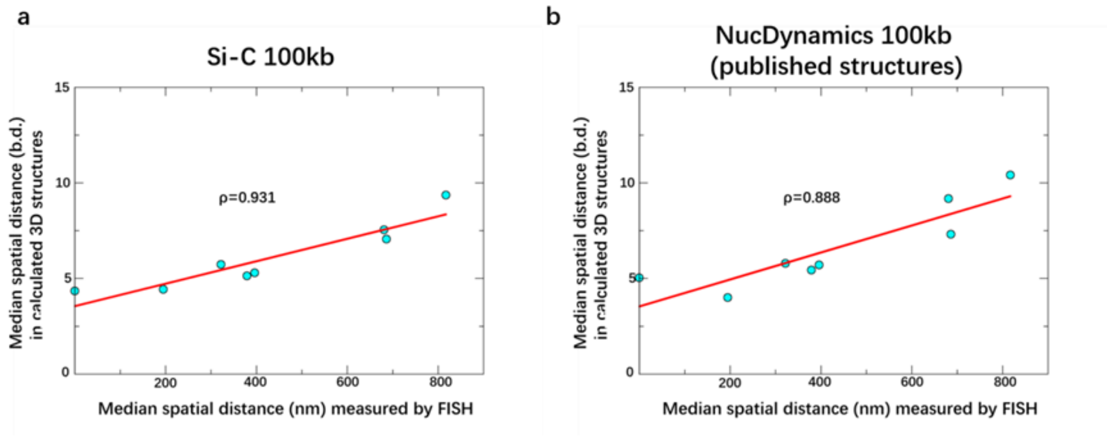

Supplementary Fig. 1: Correlation between the median spatial distances measured by eight 3D FISH probe pairs (from ref. 2) and the median distances of the corresponding pairs in the Si-C 100-kb structures (a) and in the published NucDynamics 100-kb structures (b).

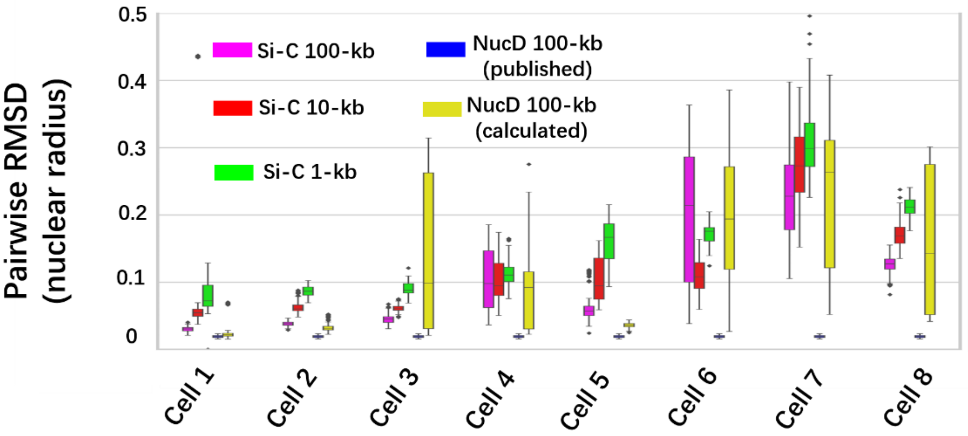

Supplementary Fig. 2: Comparison of pairwise RMSD for different structure ensembles. Boxplot of pairwise RMSDs within the Si-C ensembles at 100-kb (purple), 10-kb (red), and 1-kb (green) resolutions along with pairwise RMSDs within the published NucDynamics ensembles (blue) [structures downloaded from GEO with accession code GSE80280] and the calculated NucDynamics ensembles (yellow). Median values are shown by black bars. Boxes represent the range from the twenty-fifth to the seventy-fifth percentile. The whiskers represent 1.5 times of the inner quartile range. For each structure ensemble generated by Si-C, 20 structure replicas are used in the statistical analysis. For structure ensemble downloaded from GEO, 10 structure replicas are used. For calculated NucDynamics ensemble, 20 structure replicas are used

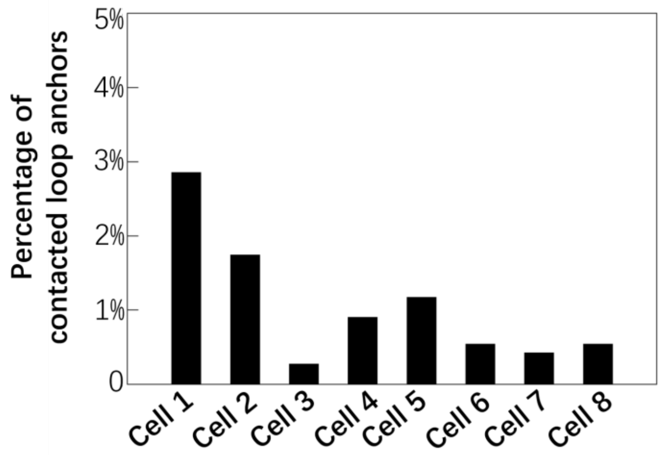

Supplementary Fig. 3: Plot of the percentage of loops that are formed in the Si-C inferred

structures of 10-kb resolution. The information of the loops is obtained from the published data (data source is listed in Supplementary Note 11).

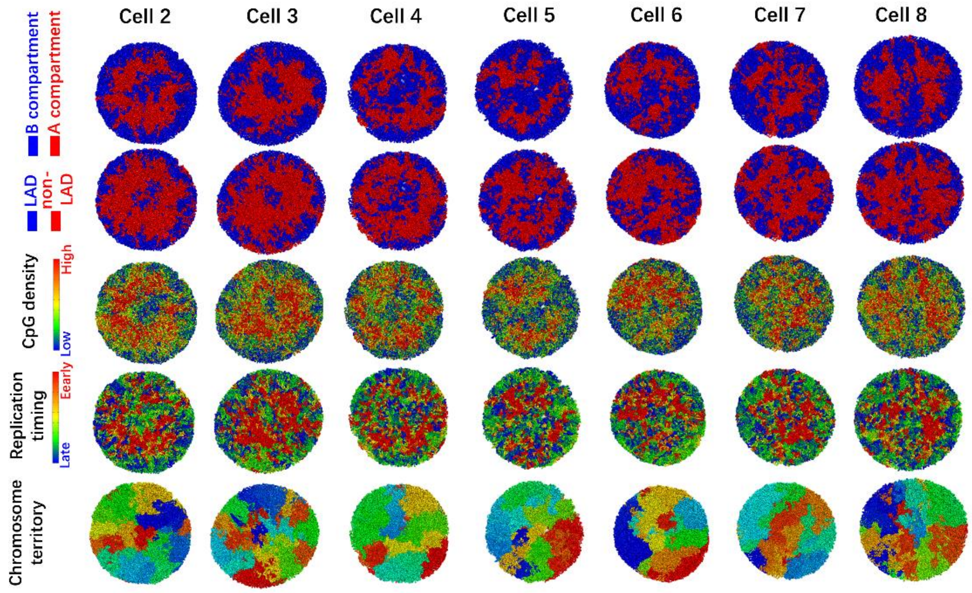

Supplementary Fig. 4: Cross-sections of the Si-C intact genome 3D 10-kb resolution structures of Cell 2-8. Colored according to whether the sequence is in the A (red) or B (blue) compartment (first column); whether the sequence is part of a lamina associated domain (LAD) (blue) or not (red) (second column); the CpG density from red to blue (high to low) (third column); the replication time in the DNA duplication process from red to blue (early to late) (fourth column).

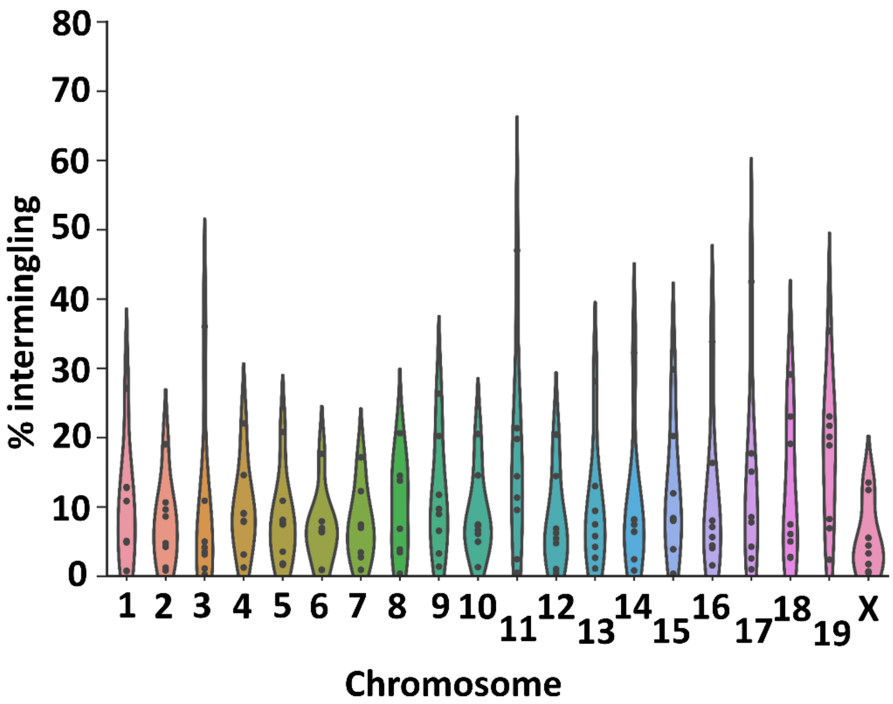

Supplementary Fig. 5: Violin plot showing the proportion of each chromosome that intermingles with other chromosomes. The proportion is derived from the Si-C 10-kb resolution structures of the eight G1-phase ES cells,.

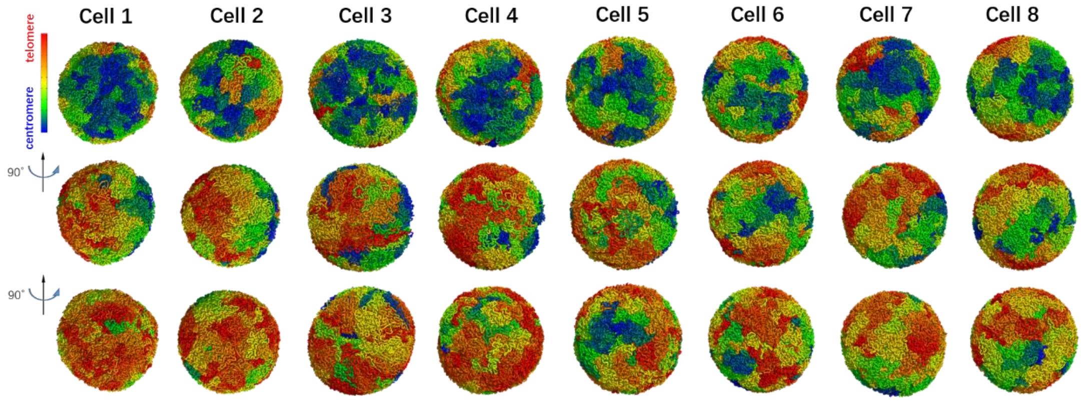

Supplementary Fig. 6: The locations of centromeres and telomeres in the Si-C intact genome 3D 10-kb resolution structures for the all eight individual cells. A consistent Rabl configuration (with centromeres and telomeres clustered on opposite sides of the nucleus) are shown in all G1-phase ES cells, strongly validating the Si-C 10-kb resolution structures.

Supplementary References

1. Theobald, D.L. Rapid calculation of RMSDs using a quaternion-based characteristic polynomial. *Acta Crystallogr A* **61**, 478-480 (2005).
2. Beagrie, R. A. *et al.* Complex multi-enhancer contacts captured by genome architecture mapping. *Nature* **543**, 519-+, doi:10.1038/nature21411 (2017).
3. Shin, H.J. et al. TopDom: an efficient and deterministic method for identifying topological domains in genomes. *Nucleic Acids Res* **44** (2016).
4. Lieberman-Aiden, E. et al. Comprehensive Mapping of Long-Range Interactions Reveals Folding Principles of the Human Genome. *Science* **326**, 289-293 (2009).
