# Supplementary material for: Si-C is a method for inferring super-resolution intact genome structure from single-cell Hi-C data
Source: Nat Commun. 2021 Jul 16;12:4369. doi: 10.1038/s41467-021-24662-z (PMC8285481; doi:10.1038/s41467-021-24662-z)
Supplement: Supplementary file 3 — Reporting Summary [file 41467_2021_24662_MOESM3_ESM.pdf]

nature research

Corresponding author(s): Luming MENG
Last updated by author(s): Jun 4, 2021

Reporting Summary

Nature Research wishes to improve the reproducibility of the work that we publish. This form provides structure for consistency and transparency in reporting. For further information on Nature Research policies, see our Editorial Policies and the Editorial Policy Checklist.

Statistics

For all statistical analyses, confirm that the following items are present in the figure legend, table legend, main text, or Methods section.

n/a	Confirmed	
☐	☒	The exact sample size (*n*) for each experimental group/condition, given as a discrete number and unit of measurement
☐	☒	A statement on whether measurements were taken from distinct samples or whether the same sample was measured repeatedly
☒	☐	The statistical test(s) used AND whether they are one- or two-sided<br>*Only common tests should be described solely by name; describe more complex techniques in the Methods section.*
☐	☒	A description of all covariates tested
☒	☐	A description of any assumptions or corrections, such as tests of normality and adjustment for multiple comparisons
☐	☒	A full description of the statistical parameters including central tendency (e.g. means) or other basic estimates (e.g. regression coefficient) AND variation (e.g. standard deviation) or associated estimates of uncertainty (e.g. confidence intervals)
☒	☐	For null hypothesis testing, the test statistic (e.g. *F*, *t*, *r*) with confidence intervals, effect sizes, degrees of freedom and *P* value noted<br>*Give P values as exact values whenever suitable.*
☐	☒	For Bayesian analysis, information on the choice of priors and Markov chain Monte Carlo settings
☒	☐	For hierarchical and complex designs, identification of the appropriate level for tests and full reporting of outcomes
☐	☒	Estimates of effect sizes (e.g. Cohen's *d*, Pearson's *r*), indicating how they were calculated

Our web collection on statistics for biologists contains articles on many of the points above.

Software and code

Policy information about availability of computer code

Data collection	No software is used in data collection
Data analysis	Using liftover code to convert reference genome from NCBI37/mm9 to GRCm38/mm10<br>Using TopDom code to identify the TAD boundaries in populated Hi-C data<br>The code of Si-C method is available on https://github.com/TheMengLab/Si-C/tree/master/Si-C_code<br>RMSD calculation https://github.com/TheMengLab/Si-C/tree/master/analysis/structure_analysis/analysis/align/rmsd<br>Separation score calculation https://github.com/TheMengLab/Si-C/tree/master/analysis/structure_analysis/analysis/align/sepscore_gyr<br>Identifying domain boundaries in single cells https://github.com/TheMengLab/Si-C/tree/master/analysis/structure_analysis/analysis/align/sepscore_gyr/boundary_chr<br>Identify chromosome compartment https://github.com/TheMengLab/Si-C/tree/master/analysis/compartment

For manuscripts utilizing custom algorithms or software that are central to the research but not yet described in published literature, software must be made available to editors and reviewers. We strongly encourage code deposition in a community repository (e.g. GitHub). See the Nature Research guidelines for submitting code & software for further information.

Data

Policy information about availability of data

All manuscripts must include a data availability statement. This statement should provide the following information, where applicable:

- Accession codes, unique identifiers, or web links for publicly available datasets
- A list of figures that have associated raw data
- A description of any restrictions on data availability

Calculated 3D structures by Si-C are presented at https://github.com/TheMengLab/Si-C_3D_structure. 3D structures generated by Nucdynamics are presented at https://github.com/TheMengLab/Nuc_3D_structure_Cell1.

Field-specific reporting

Please select the one below that is the best fit for your research. If you are not sure, read the appropriate sections before making your selection.

☒ Life sciences ☐ Behavioural & social sciences ☐ Ecological, evolutionary & environmental sciences

For a reference copy of the document with all sections, see nature.com/documents/nr-reporting-summary-flat.pdf

Life sciences study design

All studies must disclose on these points even when the disclosure is negative.

Sample size	8 cell samples are used in this work because only single-cell Hi-C data of these 8 cells are deposited in Laue's work.
Data exclusions	No data is excluded in our analysis.
Replication	For each cell, we constructed 20 3D structure replicas for different resolution. All of these structure replicas satisfy >99% constraints derived from Hi-C data. The chromosome structures exhibited by different replicas are highly consistent.
Randomization	From single-cell Hi-C data of each cell, we calculated 20 structure replicas with initial structure randomly generated using self-avoidance random walk algorithm.
Blinding	The investigators were blinded to group allocation while data obtaining and analysis

Reporting for specific materials, systems and methods

We require information from authors about some types of materials, experimental systems and methods used in many studies. Here, indicate whether each material, system or method listed is relevant to your study. If you are not sure if a list item applies to your research, read the appropriate section before selecting a response.

Materials & experimental systems

n/a	Involved in the study
☒	☐ Antibodies
☒	☐ Eukaryotic cell lines
☒	☐ Palaeontology and archaeology
☒	☐ Animals and other organisms
☒	☐ Human research participants
☒	☐ Clinical data
☒	☐ Dual use research of concern

Methods

n/a	Involved in the study
☒	☐ ChIP-seq
☒	☐ Flow cytometry
☒	☐ MRI-based neuroimaging
